# More than a medical condition: a theoretical exploration of infertility’s hidden burden

**DOI:** 10.1186/s12889-026-28050-4

**Published:** 2026-06-19

**Authors:** Pramana Adhikari, Mahesh Bhatta

**Affiliations:** 1 Public Health Officer, Silichong Rural Municipality, Sankhuwasabha, Koshi Province Nepal; 2 Public Health Officer, Health Office Sankhuwasabha, Khandbari, Koshi Province Nepal; 3 Central Department of Public Health, Institute of Medicine, Tribhuvan University, Maharajgunj, Kathmandu Nepal; 4 Central Department of Public Health Administration, Faculty of Management, Tribhuvan University, Kirtipur, Lalitpur, Nepal

**Keywords:** Infertility, Assisted Reproductive Technologies (ART), Stress and Coping Strategies, Stigma, Intersectionality, Financial burden, Psychosocial impact

## Abstract

**Background:**

Beyond its biological aspects, infertility interacts with systemic injustices, social stigma, and psychosocial pressures. To critically examine how infertility is experienced, mediated by gendered norms, structural barriers, and identity conflicts, this study draws on theoretical perspective of infertility as a socially entrenched issue by combining various concepts.

**Methods:**

This narrative review explores the psychological aspect of infertility with the synthesis of peer-reviewed articles. Literatures are critically analyzed using three theoretical frameworks (Stress and Coping Theory, stigma theory, and intersectionality theory) to form three themes namely, psychological distress and coping, stigma and gendered identity, and intersectional injustices. Theoretical frameworks allowed for a conceptually rich interpretation of findings, stressing how cultural norms, power dynamics, and institutional exclusion impacts infertility.

**Results:**

The review reveals that infertility is commonly appraised as a threat to one’s identity, relationships, and future aspirations, often leading to intense emotional turmoil. First, stress and coping theory shed light on gender differences in coping and widespread psychological suffering. Peer support and counseling were examples of adaptive methods meanwhile; avoidance and denial were the maladaptive responses. Second, the stigma theory demonstrated how infertility diminishes gendered identities, with men associating infertility with emasculation and women as “failures.” Stigma was made worse by medical prejudices, such as racial preconceptions. Third, intersectionality theory brought to light structural injustices.

**Conclusion:**

To eliminate racist and gendered hierarchies in reproductive healthcare, future interventions must place a priority on culturally appropriate mental health assistance, policy changes (e.g., subsidized IVF), and anti-stigma campaigns.

## Introduction

Infertility refers to the condition of the male or female reproductive system characterized by the inability to conceive after 12 months or more of consistent unprotected sexual intercourse [1]. It can occur by a number of different factors such as male, female or unexplained factors and few of the causes are preventable [2]. There are basically two types of infertility; primary infertility characterized by the infertility of a couple who have never been able to conceive, whereas secondary infertility is characterized by the couple’s failure to conceive after the previous pregnancy after unprotected sexual intercourse [3].The World Health Organization’s survey estimates that around 1 in 6 adults worldwide, or 17.5% of the total population, suffer from infertility. According to the updated estimates, there is little regional variation in the prevalence of infertility. The fact that the rates are similar in high, middle, and low-income nations suggests that this is a significant worldwide health concern. The lifetime prevalence was 16.5% in low- and middle-income nations and 17.8% in high-income countries [4, 5]. Infertility is a complex issue that has a significant impact on couples’ psychological health; the emotional effects are especially subtle and frequently specific to the dynamics of each connection [6]. Increased levels of anxiety, despair, and distress are common among infertile couples, and they have a substantial impact on their mental well-being and interpersonal relationships [7, 8]. Poor well-being is very common among infertile individuals. Although females often report higher levels of distress, similar psychosocial difficulties have been documented among infertile men, with poor well-being associated with lower educational attainment, treatment failure, and unexplained infertility [9–11]. Poor well-being is very common among infertile individuals, particularly among females, those with low levels of education, those whose treatments have failed, and those whose causes are unknown [12]. A systematic review by Luk and Loke (2015), which synthesized evidence from multiple studies across diverse cultural contexts, identified the four key areas of a couple’s life that were impacted by infertility; quality of life, marital relationships, sexual relationships, and psychological well-being [13]. Using the perspectives of stress and coping theory, stigma theory, and intersectionality, this narrative review looks at the emotional, social, and financial effects of infertility in an effort to shed light on the psychosocial and structural dimensions of infertility, which are often overshadowed by a purely biomedical focus. These theoretical frameworks offer a strong basis for comprehending the many and frequently related difficulties that infertile people and couples’ encounter.

**Fig. 1:**
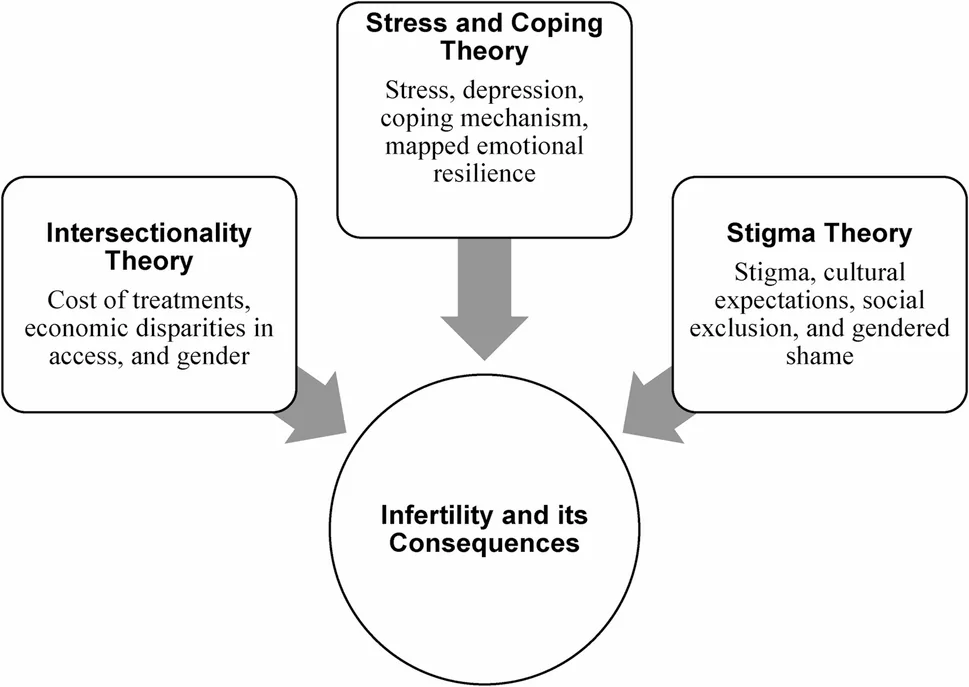
Theoretical framework and Infertility

Stress and Coping Theory developed by Lazarus and Folkman, 1984 [14] serves as a fundamental framework for comprehending how people view, react to, and cope with stress. In situations like infertility, where people deal with serious emotional, psychological, and social difficulties, the notion is especially pertinent. Stigma Theory by Goffman (1964) describes how traits that differ from social norms cause people or groups to be socially rejected. It illustrates the cultural and societal aspects of infertility, especially the ways that expectations and conventions surrounding motherhood fuel feelings of inferiority, shame, and exclusion and exclusion. Although infertility is sometimes seen as a personal battle, it is intricately woven into cultural myths that place a premium on biological motherhood [15]. The way stigma appears in various settings and how it affects relationships, self-worth, and help-seeking behaviors are all explained by this theory. Finally, intersectionality Theory by Crenshaw (1989) looks at how a person’s experiences of privilege or oppression are shaped by the intersections of many social identities, including gender, race, financial background, and culture [16]. This aids in explaining how various groups encounter stigma, mental suffering, and financial constraints associated with infertility in distinctive ways (Fig. 1). Therefore, this review seeks to explore the review question “How do psychological, social, and financial factors shape the experiences of individuals facing infertility, and how can Stress and Coping Theory, Stigma Theory and Intersectionality Theory provide a deeper understanding of these impacts?”

The PCC framework (population, concept, context) was used to define the review’s scope since it is especially well-suited for theoretical and exploratory studies that seek to synthesize evidence on experiences and perceptions rather than evaluate the impact of treatments [17].

Population: Individuals, couples facing infertility, without restriction on age, gender, cause or treatment stage.

Concept: Psychological distress, coping strategies, stigma, gendered identity, and intersectional inequities related to infertility, examined through stress and coping theory, stigma theory and intersectionality theory.

Context: Studies conducted in various cultural, economic, and healthcare settings worldwide, including high-, middle- and low-income countries.

## Methodology

This study employed a narrative review methodology to synthesize existing literature on the psychological, social, and financial aspects of infertility. A narrative review is defined as a scholarly summary that interprets and integrates findings from a body of literature to provide a comprehensive, theoretically informed overview of the study [18]. To enhance the transparency and reproducibility of the review process, structured elements were incorporated. The literature search and selection process was documented using a flow diagram adapted from the PRISMA (Preferred Reporting Items for Systematic Reviews and Meta-Analyses) [19]. While this review does not follow a systematic review protocol, PRISMA elements were adapted solely to enhance transparency of the search and selection process. Guidelines, and the methodological quality of included studies were appraised using the Critical Appraisal framework consisting of 10 questions adapted from quality assessment principles [20].

Databases such as Google Scholar, PubMed, PsycINFO, and Scopus were used in the literature search to find pertinent, peer-reviewed books, reports, and articles. The search was further refined using a variety of Boolean Operators and phrases, including “intersectionality AND reproductive health,” “infertility AND psychological impact,” “infertility AND financial burden,” “infertility AND social stigma,” and “stigma theory AND infertility.” The search results were managed and screened according to pre-defined criteria. After removing duplicate records, the titles and abstracts of the remaining articles were screened for relevance. This initial screening excluded articles that did not focus on the psychological, financial, or social aspects of infertility. The full texts of potentially relevant articles were then retrieved with the help of inclusion and exclusion criteria. Specific inclusion and exclusion criteria were used to guide the literature selection process in order to ensure legitimacy and relevance (Table 1).

**Table 1:** Inclusion and exclusion criteria for the review

Domain	Inclusion	Exclusion
Population	Individuals and/or couples experiencing infertility.	Fertile population or unrelated condition
Focus	Psychological, social, or financial aspects of infertility	Purely biomedical or clinical treatment studies
Study type	Qualitative, quantitative, mixed-methods, or review studies	Case reports, editorials, opinion pieces
Time frame	Published (2015–2025)	Published before or after this period
language	English	Non-English
Publication	Peer-reviewed literature	Non-peer reviewed sources

The Critical Appraisal Skill Program (CASP) framework was used to ensure that included studies adequately addressed key conceptual domains relevant to this review (Psychological, social, and financial aspects of infertility), thereby supporting the conceptual and content validity of the narrative synthesis. CASP informed critical interpretation but was not used to exclude or assign quality scores (Table 2). The screening questions used in quality assessment are as follows:

**Table 2:** Results of critical appraisal screening program

Studies	Q1	Q2	Q3	Q4	Q5	Q6	Q7	Q8	Q9	Q10
[16]	✔	✔	✘	✘	✔	✘	✔	✔	✘	✔
[17] London	✔	✔	✘	✘	✔	✔	✔	✔	✔	✔
[18]	✔	✔	✔	✔	✔	✔	✔	✔	✔	✔
[19]	✔	✔	✘	✘	✘	✘	✔	✔	✘	✔
[20] USA	✔	✔	✘	✘	✔	✔	✔	✔	✔	✔
[21] Turkey	✔	✔	✘	✘	✔	✔	✔	✔	✔	✔
[22]	✔	✔	✔	✔	✔	✔	✔	✔	✔	✔
[23]	✔	✔	✔	✔	✔	✔	✔	✔	✔	✔
[24]	✔	✔	✘	✘	✔	✔	✔	✔	✔	✔
[25] USA	✔	✔	✘	✘	✔	✔	✔	✔	✔	✔
[26] USA	✔	✔	✘	✘	✔	✔	✔	✔	✔	✔
[27] USA	✔	✔	✘	✘	✔	✔	✔	✔	✔	✔
[8]	✔	✔		✔	✔	✔	✔	✔	✔	✔

- Q1: Was there a clear statement of the aims of the research?
- Q2: Is the research method appropriate to address the aims?
- Q3: Was the search strategy clearly described?
- Q4: Was the selection of studies clearly described?
- Q5: Was the data from the studies included adequately described?
- Q6: Was the data analysis sufficiently rigorous and transparent?
- Q7: Were the findings clearly stated and supported by evidence?
- Q8: Was the research context and settings adequately described?
- Q9: Were the inclusion and exclusion criteria stated and justified?
- Q10: Is there discussion of study limitations?

### Flow diagram of literature identification and selection process (adapted from PRISMA)

The results were thematically synthesized with a focus on the ways in which issues associated with infertility interact with variables like gender, socioeconomic status, and cultural norms. Psychological impact includes emotional distress, anxiety, depression, and coping mechanisms employed by individuals facing infertility. Social repercussions examine the impact of interpersonal connections, cultural norms, and stigma, with a focus on how infertility affects social standing and identity. The theme of financial burden looks at accessibility concerns, in terms of finance, the expense of infertility treatments, and the socioeconomic inequalities that affect reproductive healthcare. An organized framework for comprehending the experiences and management of infertility across various social and economic origins is offered by these theme categories. This review uses the theories of stress and coping (Lazarus & Folkman, 1984), intersectionality (Crenshaw, 1989), and stigma (Goffman, 1963) to interpret the results. According to the stigma theory, infertility is viewed as a social abnormality, which results in prejudice, social isolation, and internalized shame. The theory draws attention to the ways in which cultural norms influence how people see fertility, especially gendered expectations that disproportionately affect women. The theory of intersectionality sheds light on how a person’s experience with infertility is influenced by the intersections of several social identities, including gender, socioeconomic class, and ethnicity. It assists in exposing structural disparities in healthcare access as well as the exacerbated stigma experienced by vulnerable communities. The psychological reactions to infertility are the focus of stress and coping theory, which looks at how people deal with stress by using coping mechanisms, social support, and resilience-building techniques. This notion is crucial to comprehending the behavioral and emotional adjustments made by infertile people in reaction to their difficulties.

Through the integration of diverse theoretical viewpoints, this review provides a multifaceted and complex understanding of infertility. It explores how social, psychological, and economic aspects influence the experiences of individuals dealing with infertility, going beyond medical reasons. By focusing on the need for policies and interventions that address not only reproductive health but also mental health, social inclusion, and financial accessibility, this theoretically grounded approach guarantees that the findings contribute to a more inclusive and thorough discussion on infertility.

## Findings

The findings of this review are categorized under the three themes namely, psychological distress and coping strategies, social stigma and gendered Identity, and intersectional inequities. Key information was extracted from the final set of included studies and organized into (Table 3), detailing authors, publication year, country, methodology, and key findings relevant to the review themes.

**Table 3:** Key characteristics of included studies

Author, Year, and Country	Title of the Study	Study Type	Potential Theoretical Alignment	Key Findings
Ara et al. 2022 [21]	Psychic Consequences of Infertility on Couples: A Short Commentary	Global (Commentary)	Stress and coping theory and stigma theory	When under a lot of social pressure, infertile couples may hide the issue due to the subject’s severe seclusion. The results of the medical treatment for infertility may be impacted by the discomfort and nervousness experienced throughout the procedure. Sexuality and relationship problems may exacerbate the serious narcissistic damage caused by failed attempts.
(Arya & and Dibb, 2016) [23] London	The Experience of Infertility Treatment: The Male Perspective	Qualitative Study	Stress and coping theory and stigma theory	The majority of participants talked about how they felt less of a man because they were infertile. Lack of acknowledgement that men experienced during the infertility journey from both family members and medical professionals. Due to feelings of kinship and the conviction that the kid would not be, in their judgment, their own child, several participants opposed the use of adoption and sperm donation.
(Sharma & Shrivastava, 2022) [25]	Psychological Problems Related to Infertility	Review Article	Stress and Coping theory, and Intersectionality	After starting infertility treatments, couples often discuss how they no longer have a fulfilling sexual life. “Sex on demand,” which is planned based on monthly cycles, changes from being perceived as a potentially impromptu occurrence to being seen as a chore to complete. IVF failure in the first round may force couples to choose a second round, which not only puts a strain on financially strapped couples but also has an adverse effect on mental health.
(Thoma et al., 2021) [22]	Biological and Social Aspects of Human Infertility: A Global Perspective	Book	Stress and Coping theory, and Intersectionality	Women frequently shoulder the social burden of infertility, even though men and women are equally likely to experience it. This is especially true in cultures where a woman’s identity and social worth are strongly correlated with her capacity to conceive. Due to the high expense and restricted geographic availability of diagnostic services and assisted reproductive technologies, there are still gaps in access to infertility treatment between high- and low-income groups. Obstacles to obtaining quality fertility care, a lack of political will and attention to this issue, and difficulties in comprehending the global epidemiology of infertility, including its causes and determinants, are impeding progress on infertility as a global concern in the field of sexual and reproductive health and rights.
(Öztürk et al., 2021) [26] USA	“The worst time of my life”: Treatment-related stress and unmet needs of women living with infertility	Mixed methodology	Stress and coping theory, and intersectionality theory	Infertility treatment-related stress was reported by study participants in large amounts and from a variety of sources. Due to financial constraints, geographic inequalities, and healthcare professional characteristics such unfavorable attitudes, poor communication, and ignorance of psychosocial needs, they frequently encountered significant obstacles to receiving treatment (often for years). In search of more sympathetic and encouraging care, almost half of participants had switched providers at least once.
(Ergin et al., 2018) [34] Turkey	Social stigma and familial attitudes related to infertility	Prospective Study	Stigma theory	The perception of social exclusion was present in 38% (*p* < 0.001) of infertile participants, more significantly in female partners (43% in females; 29% in males) (*p* = 0.013). 60% of the infertile participants thought that they would achieve a notable place in community after having a baby (*p* < 0.001). He community members’ attitudes were mostly negative to infertile females compared to males (57% vs. 37%; *p* = 0.001). Approximately one third (35%) thought that having a child was a must for being a family (*p* < 0.001).
(Rooney & and Domar, 2018) [24]	The relationship between stress and infertility	Review Article	Stress and coping theory	It is evident that infertility generates stress since women who experience infertility report higher levels of anxiety and despair. The most recent studies have shown that psychological therapies are effective in reducing psychological distress and are linked to notable increases in pregnancy rates.
(Gerrits et al., 2023) [28]	Breaking the silence around infertility: A scoping review of interventions addressing infertility-related gendered stigmatisation in low- and middle-income countries.	Scoping Review	Stress and coping theory Stigma theory	Several interpersonal and intra-level initiatives (such as support groups, phone hotlines, and counseling) that help men and women deal with and lessen the stigma associated with infertility. Few treatments tackled structural stigmatization (e.g., encouraging infertile women to become financially independent).
(Brown, 2022) [29] USA	Exploring the association between female infertility stigma, women’s cognitions, and coping responses	Quantitative Study	Stress and coping theory, and stigma theory	According to the findings, women who expressed more shame around infertility also had higher rates of cognitive distortions. The findings showed that infertility stigma declined with increasing self-efficacy, which could influence negative thought patterns.
(Zou et al., n.d.) [30] USA	“My Body is Betraying Me”: Exploring the Stigma and Coping Strategies for Infertility Among Women Across Ethnic and Racial Groups	Qualitative Study	Stress and coping theory, stigma theory and intersectionality theory	Results show that women mostly use the three main categories identified by the SMC model accepting, avoiding, and dismissing to cope with stigmatization. Conversely, the tactics of avoiding and rejecting accountability are noticeably less common, which reflects a larger internalization of personal accountability in the management of infertility among women.
(Pinzon et al., 2022) [35] USA	A Qualitative Exploration of Social Support in Males and Females Experiencing Issues With Infertility	Qualitative study	Stigma theory and intersectionality theory	In particular, the survey discovered that the term “loneliness and/or isolation” was frequently and candidly mentioned, especially by female respondents (20%). This was observed even in situations where caring folks and a network of support people surrounded. The most often mentioned factor overall (16%) was that those who have never been infertile cannot and do not fully comprehend the difficulties. Numerous participants expressed significant advantages and assistance from people who had similar experiences or from infertility-focused therapists. Results indicate that males with male-factor infertility (MFI) are often stigmatized.
(Collins, 2019) [27] USA	The Impact of Infertility on Daily Occupations and Roles	Qualitative transcendental phenomenological approach	Stress and coping theory, stigma theory and intersectionality theory	Being infertile can significantly affect a person’s roles and day-to-day activities. People find it challenging to maintain their regular routines and occupations, including self-care, employment, and leisure activities, due to the costs and time commitment involved in investigating fertility therapies. Along with the psychological effects of infertility, like depression, several participants reported feeling less motivated and driven to finish everyday chores like cleaning and taking care of themselves.
(Regmi et al., 2024) [8] Nepal	Quality of Life and Its Determinants Among Infertile and Non-infertile Women: A Case-control Study in Gandaki Province, Nepal	Case-control	Stress and coping theory and intersectionality theory	The average perceived stress score was significantly higher among infertile women (28.9 ± 4.61) compared to non-infertile women (25.27 ± 3.36). Similarly, mean anxiety scores were elevated in the infertile group (8.71 ± 3.00) relative to the non-infertile group (7.78 ± 2.89). Average depression scores also followed this pattern, with infertile women reporting higher levels (8.14 ± 2.67) than their non-infertile counterparts (6.86 ± 2.49).

### Psychological distress and coping strategies

Both men and women experience severe psychological suffering because of infertility, which is characterized by feelings of failure, low self-esteem, anger, worry, and sadness [21]. Despite men experiencing similar levels of mental suffering when male-factor infertility is prevalent, women typically report higher amounts [22], for example, a quantitative study in Nepal used validated scales to measure perceived stress, anxiety, and depression, finding significantly higher scores among infertile women compared to non-infertile controls [8]. Identity loss, interpersonal stress, and clinical depression levels similar to those experienced by individuals with serious illness like cancer or heart disease are frequently brought on by infertility [21, 22]. Likewise, a qualitative study also highlights the profound emotional toll and the need for more inclusive mental health support by revealing that men also experience severe psychological distress during infertility including sadness, isolation, shame, and even in very few cases suicidal thoughts [23]. Infertility treatment exacerbated by a lack of emotional and educational support and is further exacerbated by past miscarriages, financial burden, and recurrent failures [24, 25]. During fertility treatments, women suffered from severe psychological distress brought on by uncertainty, financial and emotional strain, and other pressures in their lives [8]. Hormonal drugs added to these sufferings by creating physical discomfort, emotional fluctuations, and sleep problems that made the process difficult to manage. Additionally, not addressing the psychosocial and informational need of the patients further compounded their stress; across-sectional study of 150 women in Turkey found many patients changing their health providers at least once [26]. Many infertile individuals who reported psychiatric symptoms had significantly high level of anxiety and depression than in fertile individuals [24]. Deep psychological discomfort can result from infertility because unfulfilled aspirations and emotional tension force people to retreat socially, which leads to loneliness and the loss of important relationships [27].

Effective coping mechanisms for infertility include emotional support, treatment education, and psychological therapy that target stress, anxiety, and depression. Self-administered screening tools, grief processing, and customized counselling assist individuals and couples in coping with their sorrow, accepting infertility, and considering other parenting options such as adoption [21, 25]. Through counselling and online support groups, women were able to manage their infertility and find emotional relief, empathy, and common experiences that lessened stigma and loneliness. Despite the cost of counseling, peer and professional forums provide essential opportunities for sharing concerns, learning new information, and reviving optimism [26, 28]. Psychosocial therapies, such as group-based programs like ‘The Mind Body Program’ (a structured intervention combining cognitive-behavioral therapy, relaxation techniques, and social support), improve emotional well-being, lessen distress, and in certain situations, increase the likelihood of pregnancy helping women deal with infertility [24]. In a narrative review synthesizing clinical and observational studies evaluated ‘The Mind Body Program’, a structured 10-session intervention combining cognitive-behavioral therapy, relaxation techniques, and social support. The study found that throughout treatment, women were able to regulate their negative thoughts and emotions with the help of techniques included in the program [24]. While avoidant techniques like denial and rumination exacerbate psychological discomfort, coping strategies including active coping, goal adjustment, normalization, and self-efficacy assist women in managing the stress associated with infertility. Maintaining daily routines and pursuing alternate life goals enhance wellbeing, underscoring the need for adaptable thinking and emotional control in coping with infertility and its stress [29]. Infertile women frequently resorted to coping mechanisms such as avoidance, rumination, and resignation, particularly in the absence of support. Others reclaimed their empowerment by embracing resilience via advocacy, community, faith, and self-care. A qualitative study in the United States found that coping was greatly impacted by cultural beliefs; Hispanic women frequently hid their suffering, but some African American and white women relied on their communities or directly confronted stigma [30].

### Social stigma and gendered identity

Significant societal stigma is frequently attached to infertility, and it has a dramatic effect on gendered identity, especially for men who may equate virility with self-worth, which can result in feeling of inadequacy, guilt, and sexual dysfunction. Infertility in women can restrict sexuality to a method of reproduction, which lowers enjoyment and self-esteem and increases marital conflict and worry [21]. Men’s experiences with infertility are shaped as they absorb cultural standards that associate masculinity with fertility, which causes them to self-stigmatize and be reluctant to talk about their difficulties. They become even more alone due to the absence of familial support, which exacerbates their mental discomfort and feelings of inadequacy. Due to feelings of kinship and the conviction that the kid would not be theirs in their judgement, several participants opposed the use of adoption and sperm donation. This demonstrates the profound impact that societal norms surrounding manhood have on men’s social and psychological reactions to infertility [23]. However, the relationship between masculinity and infertility is not uniform across cultures. In contexts where virility is strongly tied to patrilineal succession (e.g., parts of South Asia and sub-Saharan Africa), infertility may produce more severe emasculation stigma than in settings with more egalitarian gender norms [31, 32]. Conversely, in some western contexts, men may buffer stigma through alternative masculine identities, such as provider roles or emotional resilience [33]. Especially for women, infertility is frequently viewed as a personal failure, which increases the likelihood of self-blame, acceptance of divorce, and reluctance to report the condition. Although women are more likely to confide their spouses, men nevertheless internalize stigma and link infertility to a loss of masculinity, but the expression of this stigma varies culturally [32, 34]. Many infertile individuals thought of themselves as isolated in public and believed they had lost social value due to perceived exclusion, highlighting that psychological suffering is exacerbated by society’s heavy emphasis on having children [34].

Infertility experiences are shaped by patriarchal norms that equate childbirth with womanhood causing women in low-and middle-income countries (LMICs) to be disproportionately criticized and shunned. Because male infertility is mistakenly confused with impotence, men who are also stigmatized, frequently experience internalized shame associated with emasculation [8]. While women face social rejection, unstable marriages and sometimes violence, men are discouraged from obtaining fertility care due to societal pressures that equate masculinity with self-sufficiency and discourage help-seeking by the same gendered dynamics [28, 35]. Our review focuses primarily on psychosocial outcomes; however, we note that physical violence, reported by women in several LMIC studies [28, 35] represents a separate and urgent form of harm that warrants dedicated attention beyond the scope of this article. Perceived as a hidden stigma, infertility causes internalized guilt, feelings of deficiency, and hypervigilance in social situations. Men are similarity stigmatized by ideas of virility, but women are disproportionately affected since infertility calls into question their fundamental identity and increases social scrutiny. These gendered differences exacerbate suffering for women dealing with infertility because they are a reflection of ingrained standards that place a premium on fertility when determining a woman’s value [29]. Self-stigma associated with infertility manifests as thoughts of being “broken” or betraying one’s partner, as women internalize it as a failing of femininity. Even when male factors (i.e., infertility attributable to sperm abnormalities, hormonal dysfunction, or anatomical issues in male partner) are present, medical bias and patriarchal traditions place the blame on women ruthlessly [30]. As, infertility is often reflected as a taboo subject, many individuals keep their infertile condition to themselves meaning that they do not receive the support they need even after discussing their challenges with others they often felt misunderstood [27].

### Intersectional inequities

Intersectional inequities shape access to fertility care, with financial barriers disproportionately affecting marginalized groups. Systemic exclusion is caused by high treatment costs, lack of insurance, and related expenditures, especially for people with low incomes, members of racial or ethnic minorities, and those living in underdeveloped areas, These financial obstacles further restricts equal access to reproductive healthcare by exacerbating racial and gendered stigmas already in place [22, 26]. Couples who failed the first round of IVF may need to attempt another round, which not just creates a mental distress but also serves as a barrier for financially challenged couples [25].While all infertile women encounter gendered stigma that links femininity to reproduction, Black women deal with racist stereotypes that associate infertility with unhealthy behaviors, poverty, or promiscuity, while White women who delayed childbearing for professional reasons were sometimes accused of being “selfish”, a stereotype not typically applied to women of other racial backgrounds who also have careers [30]. These disparities are worsened by medical bias, as Black women report being turned down for fertility treatments because of doctor’s presumptions about their financial situation, creating class-based medical discrimination [30]. Some US women, particularly those in rural areas or with limited financial resources, experienced triple marginalization (financial limitation, remote location, and insensitive healthcare providers). Almost half of them changed providers in quest of compassionate care, a tactic unavailable to those with inadequate insurance or advocacy abilities [26]. Regarding LMICs, it was found that the structural factors like women’s economic dependence on their husbands or male partners were rarely addressed by LMIC-based interventions, leaving the underlying causes of vulnerability unabated [36]. Due to healthcare systems that were built with women patients in mind, while men with male-factor infertility in the UK were invisible to both their families and the medical community [23]. It was also observed that financially strapped couples are forced into debt-fueled repeat cycles when IVF fails, demonstrating how treatment practices and class interact to exacerbate pain. Furthermore, time and cost commitments of fertility therapies disrupts employments and selfcare activities (e.g., adequate rest, nutrition, exercise, and stress management), disproportionately making it difficult for working class women who cannot comply with lost wages [27]. When taken as whole, these results show that infertility is a collection of structurally placed experiences influenced by the interaction of gender with race, class, region, marital status, and the structure of the healthcare system rather than a single severe experience.

## Discussion

The results of this review can be thoroughly analyzed using the perspectives of intersectionality theory, stigma theory, and stress and coping theory, all of which together explain the complex experiences of infertility. Stress and Coping theory (Lazarus & Folkman, 1984) describes how infertility is viewed as a significant stressor that causes emotional reactions including depression, anxiety, and identity loss under the theme of Psychological Distress and coping strategies. Women increased psychological discomfort is consistent with gendered cultural norms that link parenthood to femininity, which magnifies their feelings of failure and self-blame. Many studies found that men also experience substantial suffering related to infertility, despite facing different forms of stigma and social pressure; however, men tend to underreport due to social norms that link virility and masculinity [31, 33, 37]. which reflects the focus on stigma theory on internalized shame. While avoidant coping methods like denial worsen discomfort, adaptive coping mechanisms that reduces stress are in line with coping strategies including behavioral treatments, peer-support, and counseling [38, 39]. With women more inclined to seek out social support and men retreating out of fear of being emasculated [40–42]. These gendered disparities in coping demonstrate how stigma and societal norms influence reactions to infertility.

The Social Stigma and Gendered Identity theme is deeply rooted in Stigma theory (Goffman, 1963), which pinpoints how infertility disrupts socially constructed identities. Because patriarchal standards link women’s value to their ability to procreate, they are disproportionately stigmatized, which can result in marital discord, social rejection, and feelings of being incomplete [43]. Men, on the other hand, avoid disclosure and feel alone because they absorb stigma as a danger to manhood [32]. The dual function of stigma as an internalized identity threat and a social judgment is reflected in these processes. These experiences are further contextualized by intersectionality theory (Crenshaw, 1989), which shows how stigma increases for oppressed groups. In low- and middle-income countries (LMICs), for instance, women face violence and social exclusion, while men mistake infertility for impotence, which discourages them from seeking therapy because infertility is taboo, people are kept in the dark, alone, and have less access to help, which exacerbates psychological suffering [28, 44–46]. Intersectionality Inequities are illuminated by intersectionality theory that shows how overlapping systems of oppression such as race, class, and gender shapes access care. Low-income, racialized, and rural people are disproportionately excluded from fertility treatments due to financial constraints, which compounds stigma and stress [47–50]. Systemic prejudice that intersects with gendered stigma is reflected in medical biases, such as presumptions that attribute white women’s job choices to “Selfishness” or Black women’s infertility to stereotypes about promiscuity. Cultural conventions seed these disparities; For example, African American women deal with racism and gendered expectations, while Hispanic women may hide their difficulties [30].

According to a synthesis of these views, infertility is a psychological experience influenced by identity conflicts, structural injustices, and societal norms rather than just a medical problem. Intersectionality theory reveals how overlapping oppressions lead to stratified access to care and support. Stigma theory explores the societal value of infertile identities, and stress and coping theory explains personal emotional reactions. To assist a range of infertility experiences, they collectively highlight the necessity of comprehensive interventions that address mental health, eliminate stigma, and address systemic injustices.

**Fig. 2:**
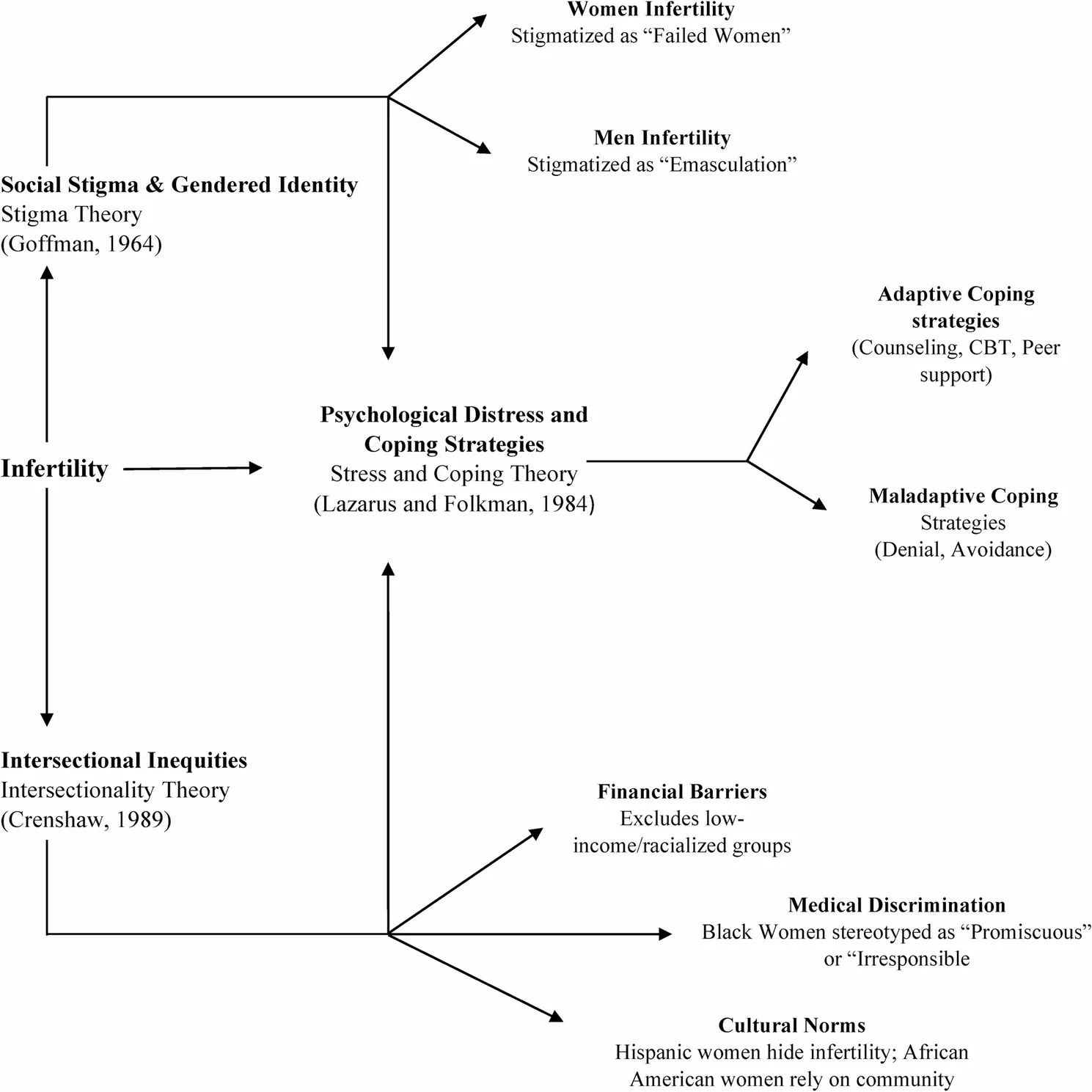
Theoretical guide in infertility experiences

The interdisciplinary theoretical framework (stress and coping, stigma, and intersectionality theories) of this theoretically informed narrative review is one of its main advantages. It offers a thorough grasp of the psychosocial aspects of infertility (Fig. 2). The integration of various qualitative and quantitative research improves the breadth of the study, and the emphasis on structural injustices draws attention to systemic obstacles that are frequently disregarded.

While this review focuses on the psychosocial and structural burdens of infertility from the individual’s perspective, it is important to acknowledge that these experiences are embedded within broader ethical, regulatory, and clinical contexts. In addition to the disparities reported in our findings, the quick development of assisted reproductive technologies, such as third-party reproduction, uterine transplantation, and pre-implantation genetic diagnosis raises difficult issues regarding access, consent, and equity [51, 52]. For example, how the challenges of ART in resource-constrained environments, cost availability, and cultural acceptance exacerbate the suffering of infertile people in LMICs, so confirming our conclusion that one of the main dimension of exclusion is financial [51]. Similarly, the stigma that infertile people face is shaped by larger cultural arguments about kinship and identity, which are reflected in ethical discussions around donor conception and the rights of offspring [52]. Future studies should look at how professional standards and regulatory frameworks, such as providing education on reproductive ethics, might either lessen or make the disparities we’ve found worse. Developing really comprehensive infertility policy and care will need integrating these medico-legal and ethical aspects with patient-centered.

Future research should use systematic review approach to lessen selection bias and broaden the interdisciplinary theoretical framework to include under-represented groups such as LGBTQI + and non-western communities. While mixed-methods techniques may better capture intersectional disparities, multilingual study participation would improve cultural ability to be generalized in practical terms, the results should guide culturally aware mental health interventions, anti-stigma initiatives that combat medical prejudices, and legislative change that promote fair access to fertility care, especially for underserved populations where systemic and financial obstacles are most noticeable. In addition to addressing the existing gaps, this dual emphasis on theoretical expansion and practical solutions would optimize the study’s effectiveness in presenting infertility as a varied and complex psychosocial experience.

### Strengths and limitations

The strength of this review is its multidisciplinary use of intersectionality, stress and coping, and stigma theories, which offer a deeper comprehension of infertility than just biological factors. Along with CASP evaluation, the incorporation of recent and varied literature increases rigor. However, because it is a narrative review that only includes a small number of English-language studies, it may be biassed in terms of selection and have limited generalizability. Additionally, this review does not address legal and ethical dimensions of assisted reproduction (e.g., child’s best interest, parentage recognition, or second-parent adoption), as these fall outside the three specified theoretical frameworks.”

## Data Availability

No datasets were generated or analysed during the current study.

## References

[1] Infertility. https://www.who.int/news-room/fact-sheets/detail/infertility. Accessed 13 Dec 2023.

[2] Infertility - an. overview | ScienceDirect Topics. https://www.sciencedirect.com/topics/medicine-and-dentistry/infertility. Accessed 21 Mar 2025.

[3] Magdum M, Chowdhury MAT, Begum N, Riya S. Types of Infertility and Its Risk Factors among Infertile Women: A Prospective Study in Dhaka City. J Biosci Med. 2022;10:158–68. 10.4236/jbm.2022.104014.

[4] Infertility Prevalence E. 1990–2021. https://www.who.int/publications-detail-redirect/978920068315. Accessed 6 May 2024.

[5] Cox CM, Thoma ME, Tchangalova N, Mburu G, Bornstein MJ, Johnson CL, et al. Infertility prevalence and the methods of estimation from 1990 to 2021: a systematic review and meta-analysis. Hum Reprod Open. 2022;2022:hoac051. 10.1093/hropen/hoac051. 36483694 PMC9725182

[6] Yang T, Wongpakaran N, Wongpakaran T, Saeng-Anan U, Singhapreecha C, Jenraumjit R, et al. Factors Associated with Depression in Infertile Couples: A Study in Thailand. Healthcare. 2023;11. 10.3390/healthcare11142004. PMC10379005 37510445

[7] Hocaoğlu Ç. The Psychosocial Aspect of Infertility. Infertility, Assisted Reproductive Technologies and Hormone Assays. 2019. 10.5772/intechopen.80713

[8] Regmi R, Yadav DK, Tiwari S. Quality of Life and Its Determinants Among Infertile and Non-Infertile Women: A Case-Control Study in Gandaki Province, Nepal. 2024;:2024.01.23.24301664. 10.1101/2024.01.23.24301664

[9] Yopo Díaz M, Watkins L. Beyond the body: Social, structural, and environmental infertility. Soc Sci Med. 2025;365:117557. 10.1016/j.socscimed.2024.117557. 39642584

[10] Hadley RA, Mumford C, Carroll M, Wilkinson K. Reproductive capital: theoretical foundations and empirical evidence from the workplace. Front Sociol. 2025;10. 10.3389/fsoc.2025.1608368. PMC12362124 40837822

[11] Fisher J, Hammarberg K. Psychological Aspects of Infertility Among Men. Endocrinology of the Testis and Male Reproduction. Cham: Springer; 2017. pp. 1–31. 10.1007/978-3-319-29456-8_46-1.

[12] Maroufizadeh S, Omani-Samani R, Bagheri-Lankarani N, Almasi-Hashiani A, Amini P. Factors associated with poor well-being of infertile people: a cross-sectional study. Middle East Fertility Soc J. 2018;23:468–70. 10.1016/j.mefs.2018.03.006.

[13] Luk BH-K, Loke AY. The Impact of Infertility on the Psychological Well-Being, Marital Relationships, Sexual Relationships, and Quality of Life of Couples: A Systematic Review. J Sex Marital Ther. 2015;41:610–25. 10.1080/0092623X.2014.958789. 25211377

[14] Biggs A, Brough P, Drummond S. Lazarus and Folkman’s Psychological Stress and Coping Theory. The Handbook of Stress and Health. John Wiley & Sons, Ltd; 2017. pp. 349–64. 10.1002/9781118993811.ch21.

[15] Inhorn MC. Right to assisted reproductive technology: Overcoming infertility in low-resource countries. Int J Gynecol Obstet. 2009;106:172–4. 10.1016/j.ijgo.2009.03.034. 19539927

[16] Atewologun D. Intersectionality Theory and Practice. In: Oxford Research Encyclopedia of Business and Management.

[17] Hardi A, BeckerGuides. Scoping Review Guide: PCC Question Outline. https://beckerguides.wustl.edu/scopingreviews/PCC. Accessed 8 Feb 2026.

[18] Sukhera J, Narrative Reviews. Flexible, Rigorous, and Practical. J Grad Med Educ. 2022;14:414–7. 10.4300/JGME-D-22-00480.1. 35991099 PMC9380636

[19] Page MJ, McKenzie JE, Bossuyt PM, Boutron I, Hoffmann TC, Mulrow CD, et al. The PRISMA 2020 statement: an updated guideline for reporting systematic reviews. BMJ. 2021;372:n71. 10.1136/bmj.n71. 33782057 PMC8005924

[20] Long HA, French DP, Brooks JM. Optimising the value of the critical appraisal skills programme (CASP) tool for quality appraisal in qualitative evidence synthesis. Res Methods Med Health Sci. 2020;1:31–42. 10.1177/2632084320947559.

[21] Ara I, Maqbool M, Zehravi M. Psychic consequences of infertility on couples: A short commentary. Open Health. 2022;3:114–9. 10.1515/openhe-2022-0022.

[22] Thoma M, Fledderjohann J, Cox C, Kantum Adageba R. Biological and Social Aspects of Human Infertility: A Global Perspective. Oxford Research Encyclopedia of Global Public Health. Oxford University Press; 2021. 10.1093/acrefore/9780190632366.013.184.

[23] Arya ST, Dibb B. The experience of infertility treatment: the male perspective. Hum Fertility. 2016;19:242–8. 10.1080/14647273.2016.1222083. PMC5152535 27563936

[24] Rooney KL, Domar AD. The relationship between stress and infertility. Dialog Clin Neurosci. 2018;20:41–7. 10.31887/DCNS.2018.20.1/klrooney. PMC6016043 29946210

[25] Sharma A, Shrivastava D. Psychological Problems Related to Infertility. Cureus. 2022. 10.7759/cureus.30320. 36407201 PMC9661871

[26] Öztürk R, Herbell K, Morton J, Bloom T. The worst time of my life: Treatment-related stress and unmet needs of women living with infertility. J Community Psychol. 2021;49:1121–33. 10.1002/jcop.22527. 33616236 PMC8324009

[27] Collins ME. The Impact of Infertility on Daily Occupations and Roles. J Reprod Infertil. 2019;20:24–34. 30859079 PMC6386796

[28] Gerrits T, Kroes H, Russell S, van Rooij F. Breaking the silence around infertility: a scoping review of interventions addressing infertility-related gendered stigmatisation in low- and middle-income countries. Sex Reproductive Health Matters. 2023;31:2134629. 10.1080/26410397.2022.2134629. PMC9970193 36811853

[29] Brown S. Exploring the Association Between Female Infertility Stigma, Women’s Cognitions, and Coping Responses. PCOM Psychology Dissertations. 2022.

[30] Zou W, Tang,Lu. and Wallis C. My Body is Betraying Me: Exploring the Stigma and Coping Strategies for Infertility Among Women Across Ethnic and Racial Groups. Health Commun 0:1–12. 10.1080/10410236.2025.2470984 39991807

[31] Agarwal A, Mulgund A, Hamada A, Chyatte MR. A unique view on male infertility around the globe. Reproductive Biology Endocrinol. 2015;13:37. 10.1186/s12958-015-0032-1. PMC4424520 25928197

[32] Pakpahan C, Ibrahim R, William W, Kandar PS, Darmadi D, Khaerana ASTA, et al. Am I Masculine? A metasynthesis of qualitative studies on traditional masculinity on infertility. F1000Res. 2023;12:252. 10.12688/f1000research.131599.1. 37008892 PMC10050908

[33] Hanna E, Gough B. The social construction of male infertility: a qualitative questionnaire study of men with a male factor infertility diagnosis. Sociol Health Illn. 2020;42:465–80. 10.1111/1467-9566.13038. 31773768

[34] Ergin RN, Polat A, Kars B, Öztekin D, Sofuoğlu K, Çalışkan E. Social stigma and familial attitudes related to infertility. Turk J Obstet Gynecol. 2018;15:46–9. 10.4274/tjod.04307. 29662716 PMC5894536

[35] Pinzon M, Rotoli S, Pinzon M, Rotoli S. A Qualitative Exploration of Social Support in Males and Females Experiencing Issues With Infertility. Cureus. 2022;14. 10.7759/cureus.29763. PMC9621735 36340522

[36] Gerrits T, Kroes H, Russell S, van Rooij F. Breaking the silence around infertility: a scoping review of interventions addressing infertility-related gendered stigmatisation in low- and middle-income countries. Sex Reprod Health Matters. 2023;31:2134629. 10.1080/26410397.2022.2134629. 36811853 PMC9970193

[37] De Jonge CJ, Gellatly SA, Vazquez-Levin MH, Barratt CLR, Rautakallio-Hokkanen S. Male Attitudes towards Infertility: Results from a Global Questionnaire. World J Mens Health. 2023;41:204–14. 10.5534/wjmh.220099. 36047077 PMC9826912

[38] Aflakseir A, Zarei M. Association between Coping Strategies and Infertility Stress among a Group of Women with Fertility Problem in Shiraz, Iran. J Reprod Infertil. 2013;14:202–6. 24551575 PMC3911816

[39] Kyei JM, Manu A, Dwomoh D, Kotoh AM, Agyabeng K, Ankomah A. Ways of coping among women with infertility undergoing assisted reproductive technologies in Ghana. Pan Afr Med J. 2022;41:29. 10.11604/pamj.2022.41.29.31319. 35291366 PMC8895556

[40] D’Souza V, Noronha JA. Social support of infertile women seeking infertility treatment: a cross sectional study. 2014.

[41] Gradvohl SMO, Osis MJD, Makuch MY. [Stress of men and women seeking treatment for infertility]. Rev Bras Ginecol Obstet. 2013;35:255–61. 10.1590/s0100-72032013000600004. 23929198

[42] Truong LQ, Luong TB, Tran TH, Dang NH, Nguyen LH, Nguyen TT, et al. Infertility-related stress, social support, and coping of women experiencing infertility in Vietnam. Health Psychol Rep. 2022;10:129–38. 10.5114/hpr.2022.113437. 38084324 PMC10681838

[43] Taebi M, Kariman N, Montazeri PD4, Majd A. Infertility Stigma: A Qualitative Study on Feelings and Experiences of Infertile Women. Int J Fertil Steril. 2021;15:189–96. 10.22074/IJFS.2021.139093.1039. 34155865 PMC8233927

[44] Ombelet W, Lopes F, Fertility care in low, and middle income countries. Fertility care in low- and middle-income countries. Reprod Fertil. 2024;5:e240042. 10.1530/RAF-24-0042. 38833569 PMC11301530

[45] Sripad P, Desai S, Regules R, Chakraborty S, Habib H, Viloria AR, et al. Exploring experiences of infertility amongst women and men in low-income and middle-income countries: protocol for a qualitative systematic review. BMJ Open. 2021;11:e050528. 10.1136/bmjopen-2021-050528. 34789491 PMC8601060

[46] Withers M. Infertility Among Women in Low- and Middle-Income Countries. In: Kickbusch I, Ganten D, Moeti M, editors. Handbook of Global Health. Cham: Springer International Publishing; 2021. pp. 885–910. 10.1007/978-3-030-45009-0_43.

[47] Afferri A, Allen H, Booth A, Dierickx S, Pacey A, Balen J. Barriers and facilitators for the inclusion of fertility care in reproductive health policies in Africa: a qualitative evidence synthesis. Hum Reprod Update. 2022;28:190–9. 10.1093/humupd/dmab040. 34888683

[48] Hall DR, Hanekom G. Assisted reproduction and justice: Threats to a new model in a low- and middle-income country. Dev World Bioeth. 2020;20:167–71. 10.1111/dewb.12252. 31769167

[49] Morshed-Behbahani B, Lamyian M, Joulaei H, Rashidi BH, Montazeri A. Infertility policy analysis: a comparative study of selected lower middle- middle- and high-income countries. Global Health. 2020;16:104. 10.1186/s12992-020-00617-9. 33097089 PMC7583186

[50] Njagi P, Groot W, Arsenijevic J, Dyer S, Mburu G, Kiarie J. Economic costs of infertility care for patients in low-income and middle-income countries: a systematic review protocol. BMJ Open. 2020;10:e042951. 10.1136/bmjopen-2020-042951. 33172951 PMC7656943

[51] Ghoshal R. Assisted reproductive technologies: Conundrums and challenges. Indian J Med Ethics. 2018;3:95–8. 10.20529/IJME.2018.030. 29724695

[52] Zaami S, Marinelli E, di Luca NM, Montanari Vergallo G. Ethical and medico-legal remarks on uterus transplantation: may it solve uterine factor infertility? Eur Rev Med Pharmacol Sci. 2017;21:5290–6. 10.26355/eurrev_201711_13854. 29228447

